# Valence and Arousal Ratings for 420 Finnish Nouns by Age and Gender

**DOI:** 10.1371/journal.pone.0072859

**Published:** 2013-08-30

**Authors:** Carina Söderholm, Emilia Häyry, Matti Laine, Mira Karrasch

**Affiliations:** 1 Department of Psychology and Logopedics, Abo Akademi University, Turku, Finland; 2 Centre for Cognitive Neuroscience, University of Turku, Turku, Finland; University of Leicester, United Kingdom

## Abstract

Language-and culture-specific norms are needed for research on emotion-laden stimuli. We present valence and arousal ratings for 420 Finnish nouns for a sample of 996 Finnish speakers. Ratings are provided both for the whole sample and for subgroups divided by age and gender in light of previous research suggesting age- and gender-specific reactivity to the emotional content in stimuli. Moreover, corpus-based frequency values and word length are provided as objective psycholinguistic measures of the nouns. The relationship between valence and arousal mainly showed the curvilinear relationship reported in previous studies. Age and gender effects on valence and arousal ratings were statistically significant but weak. The inherent affective properties of the words in terms of mean valence and arousal ratings explained more of the variance in the ratings. In all, the findings suggest that language- and culture-related factors influence the way affective properties of words are rated to a greater degree than demographic factors. This database will provide researchers with normative data for Finnish emotion-laden and emotionally neutral words. The normative database is available in Database S1.

## Introduction

There is ample evidence to suggest that words with emotional content are processed differently than emotionally neutral words. The processing specificity for emotional content in words has been demonstrated at a behavioral level [1], in electrophysiological studies, and in functional brain imaging studies (for a review, see [2]). This effect has also been found in research on the interaction between cognitive and affective processes, for example in studies examining the effects of emotion on memory [1], [3] and word recognition latencies [1], [4].

The processing specificity for emotional content in stimuli can be regarded as a generic effect, as it has also emerged for emotion-laden pictures [5], sounds [6], and odors [7]. However, words are easy to present and enable the control for several objective psycholinguistic measures known to affect cognitive processing, such as frequency [8] and word length [9].

As evidence for the processing specificity for emotion-laden stimuli has accumulated, the need to establish normative databases for the affective properties of stimuli has arisen. The measured affective stimulus properties in such databases build upon current theories on the structure of emotion. The most commonly employed theory in this regard has been the dimensional model for emotional content in stimuli. The dimensional model was introduced in 1957, when Osgood, Suci, and Tannenbaum reported that valence (‘evaluation’) and arousal (‘activity’) emerged as affective dimensions in the semantic space in several factor analyses of verbal assessments of emotional judgments [10]. The term valence is used to indicate whether the stimulus is perceived as positive/pleasant, neutral, or negative/unpleasant, whereas the term arousal is used to indicate whether the stimulus is perceived as exciting/arousing or calming.

It is important to establish language-specific normative databases for affective word content for research purposes, because it has been shown that the semantic and affective properties of words are language- and even culture-specific [11]–[13]. Several databases containing norms for valence and arousal ratings of words have been established, the first of which was Affective Norms for English Words (ANEW) [14]. Thereafter, databases have emerged in languages such as Finnish [11], German [15]–[17], French [18], Spanish [12], [19], European Portuguese [13], and Dutch [20].

As the focus of interest, especially regarding words, has been on establishing language- and culture-specific norms, less effort has been directed at establishing norms with respect to the demographic characteristics of the raters. However, there is evidence to suggest that demographic characteristics such as age [15], [18], [21]–[22] and gender [13], [21], [23] affect the valence and arousal ratings of words. Furthermore, the age-related interaction between perceived emotional content in words and word recall from memory reported by Kensinger et al. (2002) indicates that it is pivotal to take into account the effect of key demographic features on the perception of emotional content in stimuli when studying the cognitive processing of these stimuli [3]. Kensinger et al. (2002) showed that when the ANEW ratings (by young adults) were used, there was no significant interaction between age and valence in memory performance. But when the subjects’ own affective ratings were used, older adults tended to recall more negative than positive words, whereas the reversed pattern was observed for young adults.

Aging is known to affect several aspects of emotional processing, including emotion perception [21] and subjective emotional experience [24]–[26]. A common finding is the positivity effect, which is manifested such that older adults show an increased preference for positive information relative to negative information, whereas young adults tend to prefer negative information relative to positive information [27]. In subjective emotional experience studies the positivity effect has consistently been demonstrated as a decrease in self-reported negative affect [24]–[26], which seems to level out after age 60 [24], [26]. The positivity effect could also be expected to yield an increase in self-reported positive affect, but findings have been mixed in this regard. Some studies have reported such an increase (e.g., [25]), whereas other studies have observed the reversed pattern (e.g., [28]).

In the socioemotional selectivity theory framework the positivity effect has been explained as a consequence of a motivational shift related to a change in time perspective with increasing age [29]. According to this theory, older adults become more motivated to pursue emotional satisfaction, because they expect to live for a shorter period of time than young adults do. If emotional satisfaction is to be achieved, resources should be invested into mood-enhancing information.

If the preferences in processing of emotional information change with advancing age, so should the subjective response to the emotional content in stimuli in terms of valence and arousal. The positivity effect should in this respect manifest itself such that older adults should give higher mean valence ratings overall as well as for positive and negative stimuli compared to young adults. In fact, the positivity effect has been reflected in the evaluation of valence of words, albeit not consistently. Support for the positivity effect was received for the valence ratings of positive words in a study comparing valence and arousal ratings for 200 German adjectives between young adults (aged 20 to 30 years) and older adults (aged 65 to 76 years) [15]. However, in a study comparing ratings for 835 French adjectives between young adults (aged 19 to 28 years), middle-aged adults (aged 36 to 52 years), and older adults (aged 55 to 72 years), the middle-aged adults gave more positive mean ratings than the young and the older adults [18]. Also, in a study comparing ratings for 90 German verbs between young adults (aged 18 to 27 years), middle-aged adults (aged 30 to 51 years), and older adults (aged 58 to 79 years), the older adults gave more negative ratings for negative words than the young adults, but no age-related differences were found for the positive words [22]. The mixed results on age-related differences in valence ratings of words may be due to methodological differences between the studies. First, Grühn and Smith (2008) studied the differences between two age groups [15], whereas the other studies also included a third group of middle-aged adults [18], [22]. Furthermore, the age ranges used to create the age groups have varied for each study. Second, different rating scales have been employed in the studies. For example, Keil and Freund (2009) [22] used the pictorial Self-Assessment Manikin (SAM) scale [30], whereas Grühn and Smith (2008) used a 7-point Likert-scale [15]. Third, the word stimuli have differed as to amount and word class.

Aging also affects the emotional arousal or intensity aspect of emotional processing. Age-related differences in perceived intensity of subjective emotional experience as well as in physiological arousal have been observed. Older adults have shown a decreased capacity to regulate physiological arousal [31] and to inhibit processing of high-arousing material [32]. Older adults also tend to report experiencing less intense emotion in general than young adults [33]–[34], but when asking for the immediate emotional response to an event Carstensen et al. (2000) found that negative affect triggered a more intense emotional experience in older adults [24]. However, Levine and Bluck (1997) reported that the immediate response was equally intense in older and younger adults [35]. Still, it is conceivable that these difficulties in coping with high levels of physiological and emotional arousal in older age might affect the arousal ratings of stimuli.

However, previous findings regarding age-related differences in arousal ratings of words have been variable. Gilet et al. (2012) observed that middle-aged and older adults gave higher mean arousal ratings than young adults [18]. In line with the results reported by Carstensen et al. (2000) [24], Keil and Freund (2009) found that older adults gave higher arousal ratings for negative words than young adults [22]. However, the reversed effect was observed in the study by Grühn and Smith (2008), who reported that older adults gave lower arousal ratings for negative words and higher arousal ratings for positive words than young adults [15]. Grunwald et al. (1999) found no age-related differences in arousal ratings for valenced stimuli, but older adults gave higher arousal ratings than young or middle-aged adults for emotionally neutral stimuli [21]. In addition to the methodological differences mentioned above, a reason for these variable findings may be that the arousal scale has been differently conceptualized in these studies. Keil and Freund (2009) [22] used the SAM scale [30], whereas Grühn and Smith (2008) and Gilet et al. (2012) used a 7-point Likert-scale ranging from ‘very relaxed’ to ‘very tensed’ [15], [18]. The 6-point Likert-scale used by Grunwald et al. (1999) ranged from ‘not at all intense’ to ‘very intense’ [21].

Gender-related differences have been reported in emotion perception [21] and subjective emotional experience [24]–[25], [33], [36]–[37]. The results have suggested women to be more skilled at emotional processing and more emotionally reactive and receptive than men. However, findings have been inconsistent regarding gender-related differences in subjective emotional experience. In a study by Mroczek and Kolarz (1998) women reported experiencing lower positive affect than men [25], but other studies have failed to find gender-related differences in self-reported affect (e.g., [26]).

Gender-related differences in emotional processing have been more consistently demonstrated for negative emotions [38]-[39], but taken as a whole, one could argue in favor of gender-specificity regarding preference for positive and negative material (for a review, see [40]). This gender-specificity can be observed as a preference for negative stimuli in women and a preference for positive stimuli in men. The preference for negative stimuli in women could be explained in terms of an interaction between the perceived level of arousal in negative stimuli and a stronger reactivity to emotion-laden stimuli in women. Negatively valenced stimuli have been suggested to elicit stronger general reactivity in terms of high arousal compared to positive or neutral stimuli (see [22]). Women tend to report feeling more intense emotions [24], [33] and reactions to ongoing events [37], findings that have also been reflected in stronger psychophysiological responses to emotion-laden pictures [38]. Gender-related differences in the strength and loci of neural responses to negative emotions have also been demonstrated [40]. Furthermore, brain activation studies have shown that there appear to be gender-related differences in the quality and efficiency of regulation strategies to control negative emotional reactions to stimuli both at the neural and the behavioral level (for a review, see [41]). These gender-related differences in emotional processing might also be reflected in gender-related differences in valence and arousal ratings of words. The gender-related valence preference could be expected to emerge for valence ratings. The stronger and more intense reactivity to stimuli in women might manifest as higher arousal ratings overall and particularly for valenced words.

Most normative databases for the affective properties of words offer separate norms for men and women (e.g., [12], [14]), but some authors have not reported whether gender-related differences were found in their study [14], [20]. In the studies that have examined gender-related effects on valence ratings of words, the support for a gender-specific valence preference has been inconsistent. In partial support for this contention, Bellezza et al. (1986) observed that men gave more positive mean ratings than women [23]. Evidence for women’s stronger reactivity to emotion-laden stimuli has been found in that women have given more extreme valence ratings in both ends of the scale compared to men [13], [23]. However, in some studies no gender-related difference in mean valence ratings has been observed [12], [18].

Regarding arousal ratings, some support for more intense reactions to stimuli in women has been received. Soares et al. (2012) found that women gave higher mean arousal ratings than men overall [13], and Grunwald et al. (1999) reported that women gave higher mean arousal ratings for valenced stimuli, particularly for the negative ones [21]. But again, no gender-related differences in arousal ratings emerged in the studies by Redondo et al. (2007) [12] and Gilet et al. (2012) [18]. The mixed results regarding gender-related differences in affective word ratings cannot be as readily explained by methodological differences as the mixed results on age-related effects. For example, both Soares et al. (2012) [13] and Redondo et al. (2007) [12] used translations of the ANEW stimuli and the SAM scales [30]. The only methodological difference between these studies lies in the amount of words rated by each respondent (on average 129 words in Redondo et al. (2007) [12], and 60 words in Soares et al. (2012) [13]).

The first aim of the present study was to collect valence and arousal ratings for a set of 420 Finnish nouns in order to provide age- and gender-specific normative data for these affective dimensions. To date, no such norms have been reported for Finnish words. Eilola and Havelka (2010) published norms for valence and arousal ratings, as well as for concreteness, familiarity and offensiveness ratings, for 210 Finnish nouns and their British English counterparts [11]. The valence and arousal ratings were collapsed over all participants and no age- or gender-specific norms were reported. Furthermore, their participants included only 16–45-year-olds, whereas we aimed at collecting ratings among older adults (≥ 60 years) as well. In addition, we included corpus-based frequency values in our database. The inclusion of objective psycholinguistic measures should add to the usefulness of the corpus in experimental studies, as these measures affect word processing [8]-[9]. The present word frequency values were obtained from an unpublished extensive database of written Finnish (the Finnish newspaper Turun Sanomat published between 1^st^ March 1994 and 30^th^ June 1996, including 22.7 million words) using the computerized WordMill Lexical Search Program [42].

The second aim of the study was to examine the characteristics of the word ratings in this database by analyzing (a) the distribution of the ratings in the affective space, (b) possible age-and gender-related differences in mean valence and arousal ratings for all words and for words categorized by their mean valence ratings, and (c) the relationship between the ratings in this study and the ratings in previous studies using the same words [11]-[12], [14]. The bivariate distribution of ratings on the dimensions of valence and arousal plotted in a two-dimensional space, also called the affective space, has usually shown a curvilinear shape with a predominantly quadratic trend [11]–[14], [16]–[17], [19]–[20], [22]. The curvilinear valence-arousal relationship reflects the fact that stimuli that are rated high in either positive or negative valence also elicit high arousal ratings, whereas stimuli rated as neutral in valence elicit low arousal ratings. However, the clear curvilinear shape has been evident in studies using young adult raters, whereas older adults have shown an increasingly linear relationship between valence and arousal ratings [22]. In particular, the association between valence and arousal ratings for positive words has decreased and the association for negative words increased for older adults [22]. Because we included older adult raters, we expected the quadratic trend to be less prominent than in previous studies, and the association between valence and arousal ratings for positive words to be weaker relative to that for negative words.

The choice of age and gender as variables of interest was based on previous findings indicating that these demographic characteristics affect emotional processing. Because of the mixed evidence supporting a positivity effect in valence ratings of words and an age-related effect on arousal ratings, our predictions for these effects were largely tentative. We expected older adults to give higher valence ratings for positive words compared to the younger age groups. Also, we predicted that older adults would give higher mean arousal ratings overall and for the negative words. As for gender-related effects, again because of the mixed results in previous work, our predictions were provisional. Based on the hypothesis of stronger and more intense emotional reactivity in women, we expected women to give more extreme valence ratings, especially for the negative words. Also, women were expected to give higher mean arousal ratings than men, especially for the negative words.

## Method

### Ethics Statement

The study was approved by the Institutional Review Board of the Department of Psychology (now the Department of Psychology and Logopedics) at Abo Akademi University. All participants gave written informed consent prior to entering the study. They entered the study with the understanding that participation was anonymous and that the answers would be treated confidentially. Informed consent was therefore also given anonymously by all participants, including the minors. This procedure was approved by the Institutional Review Board. Indeed, the Institutional Review Board waived the need for parental consent given that there were no privacy issues involved (all participants remained anonymous) and that participation in the study was deemed to carry only a minimal risk, i.e., no greater than they would experience in daily life or during routine psychological tests.

### Participants

A community sample of 1155 volunteers was obtained through e-mail invitations. The only inclusion criterion was Finnish as the native language. It was not possible to calculate view rates, participation rates, or completion rates as suggested by Eysenbach [43] because of the limitations of the survey software. These limitations also rendered it impossible to prevent multiple responses from the same individual and to check for completeness, resulting in missing values. One hundred fifty-nine respondents were excluded: sixty-seven respondents (5.8%) reported some other language than Finnish as their native language, forty-nine respondents (4.2%) had missing values in the demographic questionnaire part of the survey, four respondents (0.3%) had missing values in the ratings (equal to or more than 10% missing), and thirty-nine respondents (3.4%) produced ratings that were mostly outliers, *z* = + /−3.29, *p*<.001, which indicated that their evaluations had been conducted in a haphazard manner (e.g., evaluating all nouns as ’very pleasant’). The 996 respondents remaining for the statistical analyses were all native Finnish speakers, brought up in a monolingual home. The sample consisted of 754 women (75.7%) and 242 men (24.3%), ranging in age from 16 to 77 years, *M* = 32.91, *SD* = 14.50.

### Materials and Procedure

An initial list of 847 Finnish nouns was generated. Only nouns were included, because this study was conducted as part of a study on age-related changes in memory for emotion-laden nouns. The final 420 nouns (Database S1) were selected for evaluation using the following criteria. All were nouns in nominative singular, which is the morphologically simple dictionary form in Finnish. They were chosen to represent positive, negative, and neutral valence categories, using ANEW [14] as a guideline. Of the words chosen, 156 (37.1%) can be found in ANEW. All included nouns had low lexical ambiguity (homonymy) in the Turun Sanomat corpus. The selected nouns were also checked for their surface frequency and their word length in letters. The surface frequency stands for the frequency of one particular word form (e.g., *love* has a different surface frequency than *loved*). The selected 420 nouns had a surface frequency value of 0.04–84.05 per million (*M* = 8.49, *SD* = 14.99), indicating low to medium frequency. The word length of the selected nouns was limited to a range of 5 to 9 letters, *M* = 6.79, *SD* = 1.34.

The 420 Finnish nouns were pseudorandomized into four different word lists. A word list consisted of an equal number of nouns assumed to be rated as negative, neutral, and positive, resulting in 35 nouns of each assumed valence category in each list (Database S1). The pseudorandomization procedure also entailed matching the four word lists on word length in letters, *F*(3, 416) = 0.096, *p* = .962, on surface frequency, *χ* ^2^(3, *N* = 420) = 0.289, *p* = .962, and on lexical ambiguity rate, *χ^2^*(3, *N* = 420) = 4.610, *p* = .203. Each list was finally duplicated and rerandomized, resulting in a total of eight web surveys. This was done with the aim of minimizing any order or habituation effects.

The web surveys were created using the Sydaco software, a computer program maintained by the Computing Centre at Abo Akademi University at the time of data collection (Spring 2008). The first nine items were questions concerning demographic data, physical health status, and psychiatric health status. The respondents were also asked to evaluate their current level of arousal and feeling of pleasure, and their overall level of arousal and feeling of pleasure during the past four weeks on a Likert-type scale ranging from 1 to 7. The remaining 105 items were the nouns to be evaluated.

A pilot study was conducted to specify whether the survey was of appropriate length, the questions and instructions were clear, and the initial 9-point Likert-scales based on the 9-point SAM scales [30] were suitable. The results indicated that the amount of time required to complete the survey was approximately 15 minutes. The valence and arousal scales were reduced to 7-point scales, because the pilot respondents did not give any extreme ratings.

In the actual surveys, the instructions were given as follows (translated from Finnish):

“In the next part the task is to evaluate how pleasant and arousing you consider each of the following words. Evaluate the pleasantness of the word on a scale of 1–7, where 1 = very unpleasant, 4 = neutral (neither pleasant nor unpleasant), 7 = very pleasant. Evaluate the arousal of the word on a scale of 1–7, where 1 = very calming, 4 = neutral (neither calming nor arousing), 7 = very arousing. Consider each word carefully and use the whole scale when evaluating the words. Fill in every section carefully. When you have evaluated all the words, click the “send” button and the answers will be registered”.

The surveys were posted on the website of the Department of Psychology (now the Department of Psychology and Logopedics) at Abo Akademi University. An e-mail invitation with a link to one of the surveys was sent to e-mail lists at universities, vocational schools, adult education institutes, employees at the city of Turku, and different organizations for elderly adults in Finland. Additionally, the invitation was posted on two Finnish internet discussion forums for elderly adults. The different organizations were chosen with the aim of collecting data from a demographically representative sample. Before sending the invitations, authorization had been acquired from the web administrator of each organization. The mailing lists were randomly distributed between the eight surveys with the limitation that a demographically representative sample of raters would be evaluating each of the four word lists. Because the addresses to the surveys were sent to potential respondents, there were no links to them from the main page. The surveys were open for approximately one month after having sent the invitations. A voluntary lottery of two gift vouchers (value: 100€ and 50€) to a national bookstore chain was used as an incentive. The contact information for the lottery was collected separately from the word evaluations to ensure privacy. When the data had been collected, the eight web surveys were merged to correspond to the initial four word lists.

### Description of the Supplementary Material

The supplementary material is provided in Database S1. The material is organized in the file as follows.

Finnish word: The nouns listed in alphabetical order.

Engl. Transl.: English translations of the Finnish nouns. The Finnish nouns were independently translated by two of the authors (Häyry and Söderholm), proficient in both languages. The English translation has been italicized if the noun can be found in ANEW [14].

Word list: Number of word list in which the word appeared (1–4).

Affective ratings: Mean values (*M*) and standard deviations (*SD*) for valence and arousal ratings, separately for the whole sample (All), and for the demographic groups divided by gender and age.

Psycholinguistic measures, obtained by the computerized WordMill Lexical Search Program [42] from an unpublished extensive database of written Finnish (the Finnish newspaper Turun Sanomat published between 1^st^ March 1994 and 30^th^ June 1996, including 22.7 million words):

Nr letters: Word length in letters.
Surface: Surface frequency, i.e., the frequency of one particular word form.
Lemma: Lemma frequency, i.e., the summative frequency of all the word forms of a word.
For the surface and lemma frequencies, absolute and relative values (per million) are given. The relative value = (absolute value / 22,700,000) * 1,000,000.
Bigram: Bigram frequency, i.e., the average frequency of all two-letter combinations in a word.
Initrigram: Initial trigram frequency, i.e., the average frequency of all word-initial three-letter combinations.
Fintrigram: Final trigram frequency, i.e., the average frequency of the word-final three-letter combination.

## Results

### The Relationship between Valence and Arousal Ratings for All Respondents

**Figure 1:**
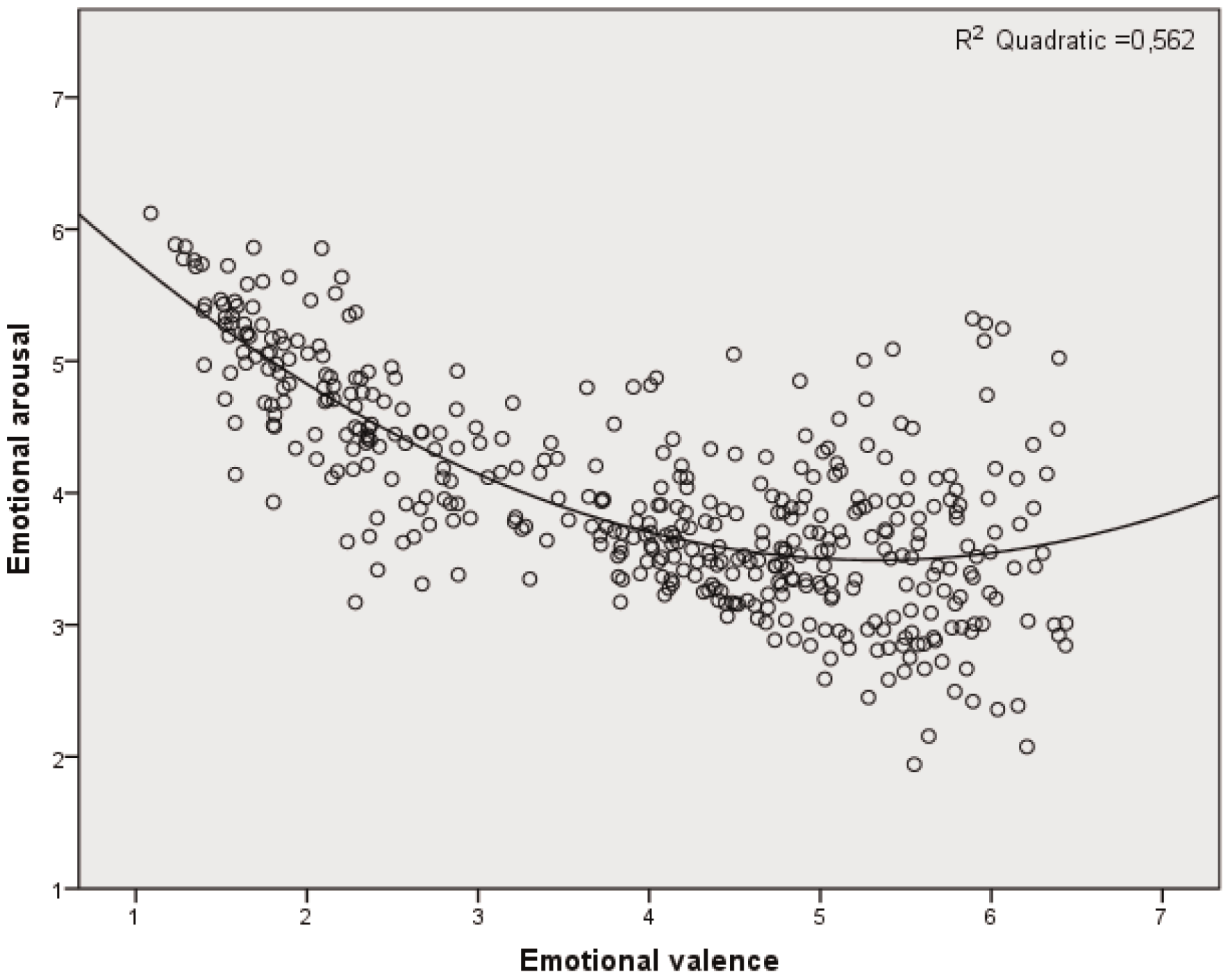
The relationship between valence and arousal ratings for each word averaged across all participants.

Figure 1 shows the distribution of the mean valence and arousal ratings in the bivariate affective space. As expected, the distribution was curvilinear, so that the nouns rated as either positively or negatively valenced were also rated as more arousing than the emotionally neutral nouns. To verify the curvilinear relationship between the two affective dimensions, we conducted a model fit analysis with mean valence rating as the independent variable and mean arousal rating as the dependent variable. A significant quadratic relationship emerged, *R* = .75, *p*<.001, explaining 56% of the variance. However, as depicted in the right-hand area of Figure 1, the arousal ratings of the positive nouns were more evenly distributed over the arousal scale compared to those of the negative nouns. This was also indicated by the beta coefficients for the quadratic relationship, such that the initial negative relationship between valence and arousal ratings, *b_1_* = −2.40, was steeper than the later positive relationship, *b_2_* = 1.72.

To explore this finding in more detail, we conducted pairwise correlation analyses separately for negative, neutral, and positive nouns. The word valence categories were created as follows: negative (mean valence rating between 1 and 3; *n* = 133); neutral (mean valence rating between 3.01 and 4.99; *n* = 156); and positive (mean valence rating between 5 and 7; *n* = 131) nouns. The pairwise correlation analyses confirmed that the negative relationship between valence and arousal ratings was strongest for the negative nouns, *r*(133) = .719, *p*<.001. Contrary to expectations, the correlation reached statistical significance for the neutral nouns, *r*(156) = −.365, *p*<.001, but not for the positive nouns, *r*(131) = .010, *p* = .910. When the nouns were classified into arousal categories using the same cut-off scores as for valence, significantly different classification patterns emerged for each valence group, *χ^2^*(4) = 154.29, *p*<.001. As can be seen in Table 1, there were no low-arousing negative nouns in this database, whereas a larger number of positive nouns were classified as low-arousing compared to the neutral nouns. Also, a larger proportion of the negative nouns were classified as high-arousing, compared to the neutral nouns and the positive nouns. In Table 1 it can also be seen that the respondents preferred the middle values on the arousal scale regardless of the valence rating of the noun.

**Table 1:** Proportion of Nouns in Arousal Groups.

	Valence						
Arousal	Negative		Neutral		Positive		Total
Low	0	(0.0%)	4	(2.6%)	37	(28.2%)	41
Intermediate	86	(64.7%)	151	(96.8%)	87	(66.4%)	324
High	47	(35.3%)	1	(0.6%)	7	(5.3%)	55
Total	133	(100.0%)	156	(100.0%)	131	(100.0%)	420

*Note.* The valence and arousal groups were created as follows: low/negative score range 1–3; intermediate/neutral score range 3.01–4.99; high/positive score range 5–7.

### Age- and Gender-Related Differences in Valence Ratings

Four age groups were created to study age-related differences in valence and arousal ratings. The four age groups consisted of adolescents (age range = 16–19), young adults (age range = 20–30), middle-aged adults (age range = 31–59), and older adults (age range = 60–77). The demographic characteristics of the four age groups are shown in Table 2.

**Table 2:** Demographic Characteristics of the Four Age Groups.

		Adolescents	Young adults	Middle-aged	Older adults
Characteristic		(16–19 yrs)	(20–30 yrs)	(31–59 yrs)	(60–77 yrs)
*n*		88	523	322	63
Age *M (SD)*		18.16 (0.99)	23.71 (2.63)	45.44 (8.27)	65.81 (4.55)
Gender	Women	65 (73.9%)	385 (73.6%)	264 (82.0%)	40 (63.5%)
	Men	23 (26.1%)	138 (26.4%)	58 (18.0%)	23 (36.5%)
Education	Low	32 (36.4%)	6 (1.1%)	24 (7.5%)	8 (12.7%)
	Average	50 (56.8%)	276 (52.8%)	156 (48.4%)	21 (33.3%)
	High	6 (6.8%)	241 (46.1%)	142 (44.1%)	34 (54.0%)

*Note.* The educational levels were created using the Unesco International Standard Classification of Education (ISCED) system, as adapted to the Finnish education system by Statistics Finland (Statistics Finland, n.d.). The low level corresponds to ISCED levels 1 and 2, the average level to ISCED levels 3 and 4, and the high level to ISCED levels 5A and 6.

Two factorial analyses of variance (ANOVA) were conducted with age group (adolescents vs. young adults vs. middle-aged adults vs. older adults), gender (women vs. men), and word valence (positive vs. negative vs. neutral words) as between-subjects factors and the mean ratings of valence and arousal for each word as dependent variables.

Table 3 presents the means for the valence and arousal dimensions calculated separately for each demographic subgroup. The factorial ANOVA with mean valence ratings as the dependent variable showed a main effect of all the independent variables: age, *F*(3, 10056)  = 3.11, *p* = .025, η_p_^2^ = .001; gender, *F*(1, 10056) = 14.46, *p*<.001, η_p_^2^ = .001; and word valence, *F*(2, 10056) = 10701.86, *p*<.001, η_p_^2^ = .680. Three significant two-way interactions were observed: an Age × Word valence interaction, *F*(6, 10056) = 24.68, *p*<.001, η_p_^2^ = .015; a Gender × Word valence interaction, *F*(2, 10056) = 58.48, *p*<.001, η_p_^2^ = .011; and an Age × Gender interaction, *F*(3, 10056) = 10.68, *p*<.001, η_p_^2^ = .003. The factorial ANOVA further showed a significant three-way Age × Gender × Word valence interaction, *F*(6, 10056) = 3.15, *p* = .004, η_p_^2^ = .002.

**Table 3:** Means for Valence and Arousal Ratings by Age and Gender.

		Adolescents			Young adults			Middle-aged adults			Older adults		
Dimension		Women	Men	All	Women	Men	All	Women	Men	All	Women	Men	All
Valence	All	3.93	4.03	3.98	4.07	3.97	4.02	4.01	3.90	3.96	4.11	3.95	4.03
	Negative	2.09	2.65	2.37	2.30	2.39	2.35	2.02	2.09	2.06	2.00	2.09	2.04
	Neutral	4.16	4.15	4.16	4.33	4.16	4.25	4.27	4.13	4.20	4.45	4.26	4.36
	Positive	5.51	5.28	5.39	5.56	5.33	5.45	5.74	5.46	5.60	5.85	5.46	5.65
Arousal	All	3.91	3.50	3.70	3.98	3.85	3.91	3.89	3.76	3.83	4.10	3.82	3.96
	Negative	4.57	3.80	4.18	4.78	4.49	4.63	4.65	4.22	4.43	4.93	4.35	4.64
	Neutral	3.63	3.29	3.46	3.72	3.64	3.68	3.56	3.46	3.51	3.84	3.53	3.68
	Positive	3.57	3.44	3.50	3.47	3.44	3.46	3.53	3.67	3.60	3.57	3.62	3.60

Follow-up analyses were conducted for a detailed analysis of the findings. Only results significant at the *p*<.01-level are reported for the follow-up analyses to correct for multiple comparisons and to minimize the risk for Type I error. The Bonferroni adjustment may be too conservative, thus increasing the risk for Type II error [44].

Naturally, all the post hoc comparisons of the main effect of word valence reached statistical significance at the *p*<.001-level. Tukey HSD post hoc comparisons for the main effect of age failed to reach statistical significance. However, the comparison between middle-aged and older adults approached significance at *p*s = .027. There was a trend indicating that older adults rated the nouns as slightly more positive than middle-aged adults (Table 3). Tukey HSD post hoc comparisons for the Age × Word valence interaction showed that older adults, as well as middle-aged adults, rated the positive nouns as more positive than adolescents, *p*s<.001, and young adults, *p*s = .003 and *p*s<.001, respectively. Also, older adults rated the neutral nouns as more positive than adolescents, *p*s<.001, and middle-aged adults, *p*s = .002. However, middle-aged and older adults, *p*s<.001, rated the negative nouns as more negative than adolescents or young adults (Table 3).

The follow-up analysis of the main effect of gender also only approached significance at *p* = .027, showing that women tended to give more positive mean ratings than men. Follow-up analyses of the Gender × Word valence interaction indicated that women rated the negative nouns as more negative, *M* = 2.10, than men, *M* = 2.30, *t*(3190) =  5.83, *p*<.001. Furthermore, women rated both the neutral, *M* = 4.30, and the positive nouns, *M* = 5.66, as significantly more positive than men, *M* = 4.18, *t*(3742) =  4.10, *p*< .001, and *M* = 5.38, *t*(3142) = 9.40, *p*<.001, respectively.

The follow-up analyses of the two-way interaction Age × Gender did not reach statistical significance. Approaching significance at *p* = .021, older women tended to rate the nouns as more positive than older men (Table 3). Follow-up analyses of the Age × Gender × Word valence interaction revealed that the negative nouns were rated as more negative by female adolescents compared to male adolescents, *t*(796) = 8.47, *p*<.001 (Table 3). Women gave significantly more positive ratings than men for the positive nouns in every age group, adolescents: *t*(784) = 3.77, *p*<.001; young adults: *t*(784) = 4.79, *p*<.001; middle aged adults: *t*(784) = 4.78, *p*<.001; older adults: *t*(784) = 5.68, *p*< .001. The neutral nouns were rated as more positive by young adult women compared to young adult men, *t*(934) = 3.64, *p*<.001, and by older women compared to older men, *t*(934) = 2.76, *p* = .006.

The strongest effect by far was the main effect of word valence with η_p_^2^  = .680. The second strongest effect was seen for the Age × Word valence interaction with η_p_^2^ = .015. This indicates that the demographic characteristics of the raters had a relatively small impact on the valence ratings for these nouns.

### Age- and Gender-Related Differences in Arousal Ratings

The factorial ANOVA with mean arousal ratings as the dependent variable showed a main effect of all the independent variables: age, *F*(3, 10056) = 29.62, *p*<.001, η_p_^2^ = .009; gender, *F*(1, 10056) = 135.12, *p*<.001, η_p_^2^ = .013; and word valence, *F*(2, 10056) = 854.48, *p*<.001, η_p_^2^ = .145. Three significant two-way interactions were observed: an Age × Word valence interaction, *F*(6, 10056) = 10.09, *p*<.001, η_p_^2^ = .006; a Gender × Word valence interaction, *F*(2, 10056) = 52.33, *p*<.001, η_p_^2^ = .010; and an Age × Gender interaction, *F*(3, 10056) = 10.90, *p*<.001, η_p_^2^ = .003.

Only findings significant at the *p*< .01-level are reported for the follow-up analyses of the main effects and the interaction effects observed for the arousal ratings to correct for multiple comparisons and to minimize the risk for Type I error. The Bonferroni adjustment may be too conservative, thus increasing the risk for Type II error [44].

In a further confirmation of the results reported above, Tukey HSD post hoc comparisons for the main effect of word valence revealed that the negative nouns received the highest arousal ratings on average, *M* = 4.47, *p*s<.001, compared to both the neutral, *M* = 3.58, and the positive nouns, *M* = 3.54, whereas the difference in arousal ratings between the neutral and the positive nouns was not statistically significant.

Tukey HSD post hoc comparisons for the main effect of age revealed that adolescents gave significantly lower mean arousal ratings than all the other age groups (*p*s< .001; Table 3), as did middle-aged adults compared to older adults (*p*s< .001). Follow-up analyses of the Age × Word valence interaction showed that adolescents rated the negative nouns as less arousing than the other age groups, *p*s<. 001, as did middle-aged adults compared to young adults, *p*s = .002, and older adults, *p*s< .001 (Table 3). Adolescents also rated the neutral nouns as less arousing than young adults and older adults, *p*s< .001, as did middle-aged adults compared to young adults and older adults, *p*s< .001. There were no statistically significant age-related differences in arousal ratings for the positive nouns.

Follow-up analyses of the main effect of gender showed that women gave significantly higher mean arousal ratings, *M* = 3.97, than men, *M* = 3.73, *t*(10078) = 10.63, *p*<.001. Follow-up analyses of the Gender × Word valence interaction revealed that women rated the negative and the neutral nouns as more arousing, *M* = 4.73; *M* = 3.69, respectively, than men, *M* = 4.21; *M* = 3.48, respectively, *t*(3190) = 12.96, *p*<.001, and *t*(3742) = 7.18, *p*<.001, respectively. There were no statistically significant gender-related differences in arousal ratings for the positive nouns.

Follow-up analyses of the Age × Gender interaction revealed that women, regardless of age group, gave higher mean arousal ratings than men, adolescents: *t*(2518) = 9.58, *p*<.001; young adults: *t*(2518) = 3.24, *p*<.001; middle-aged: *t*(2518) = 2.93, *p* = .003; older adults: *t*(2518) = 5.58, *p*<.001 (Table 3).

The strongest effect was again the main effect of word valence group with η_p_^2^ = .145. The second strongest effect was observed for the main effect of gender with η_p_^2^ = .013. This indicates that the demographic characteristics of the raters had a relatively small effect on the arousal ratings as well.

### Correlations with Valence and Arousal Ratings in Other Databases

We conducted Pearson correlation analyses between valence ratings and arousal ratings in our database and in three other databases. Our database had 54 words in common with the database established by Eilola and Havelka (2010) [11]. Valence ratings correlated strongly, *r*(54) = .99, *p*<.001, whereas the correlation between arousal ratings did not reach statistical significance, *r*(54) = .23, *p* = .102. To explore this finding in more detail, we conducted pairwise correlation analyses between arousal ratings separately for negative, neutral, and positive nouns. The correlation for the negative nouns was the only one to reach statistical significance, *r*(26) = .43, *p* = .028. The correlation for the neutral nouns was *r*(8) = .60, *p* = .116, and the correlation for the positive nouns was *r*(20) = .11, *p* = .649.

One hundred fifty-six words of those in our study could be found both in ANEW [14] and in Redondo et al. (2007) [12], which contains all the ANEW words translated to Spanish. The correlations between valence ratings were again strong, *r*(156) = .93, *p*<.001, and *r*(156) = .94, *p*<.001, respectively, whereas the arousal ratings correlated more weakly but still significantly, *r*(156) = .67, *p*<.001, and *r*(156) = .60, *p*<.001, respectively. The correlations between arousal ratings conducted separately for negative, neutral, and positive nouns were all statistically significant. The correlations with the ANEW arousal ratings [14] were *r*(51) = .75, *p*<.001 for the negative nouns, *r*(37) = .69, *p*<.001 for the neutral nouns, and *r*(68) = .80, *p*<.001 for the positive nouns. The correlations with the Redondo et al. (2007) [12] arousal ratings were *r*(51) = .68, *p*<.001 for the negative nouns, *r*(37) = .63, *p*<.001 for the neutral nouns, and *r*(68) = .63, *p*<.001 for the positive nouns.

## Discussion

The present study aimed at collecting valence and arousal ratings for a set of 420 Finnish nouns to provide age- and gender-specific normative data. We also included corpus-based frequency values in the database to add to its usefulness in experimental studies, as these objective psycholinguistic measures are known to affect word processing [8]-[9].

The second aim of this study was to describe the characteristics of the word ratings in this database by examining the distribution of the ratings in the affective space, possible age- and gender-related differences in mean valence and arousal ratings for all nouns and for nouns categorized by their mean valence ratings, as well as the relationships between ratings in our study and those in previous databases [11]–[12], [14].

As expected, the distribution of these 420 Finnish nouns in the affective space was similar to the typical curvilinear shape found in other studies [11]–[14], [16], [19], [22], with the exception of the positive nouns, the arousal ratings of which were more evenly distributed over the arousal scale. More detailed analyses showed quite unexpectedly that there was no correlation between valence and arousal ratings for the positive nouns. However, the expectation that the linear relationship between valence and arousal ratings for the negative nouns would be stronger compared to that for the positive nouns due to the inclusion of older adult raters in the sample was confirmed (cf., [22]). A reason for the zero correlation may be that there were more low-arousing nouns among the positively valenced nouns compared to both the neutral and the negative nouns. Also, the mean arousal rating for the positive nouns was lower than that for the negative or neutral nouns, which is in line with the results reported by Grühn and Smith (2008), who also included older adult raters in their sample [15].

There were decidedly more high-arousing nouns among the negative nouns compared to both the neutral and the positive nouns. The higher frequency of high-arousing words among negatively valenced words has been reported by other studies as well [13], [15]. This finding has been suggested to reflect greater general reactivity to negatively valenced stimuli (see [22]). Janschewitz (2008) put forth the idea that the enhanced arousal ratings of negative words might be a natural property of the affective lexicon [45]. In line with previous research (e.g., [11]–[12], [14]), nouns rated as neutral in valence were also rated as neutral/intermediate in arousal in our study.

However, other factors than a possible age-related effect might explain the lack of a correlation between valence and arousal ratings for the positive nouns, because our findings regarding arousal ratings for positive words are in line with previous studies using young adult raters by Ferré et al. (2012) [19] and Soares et al. (2012) [13]. Furthermore, and quite surprisingly, the correlation between the arousal ratings in our study and those for Finnish nouns in the study by Eilola and Havelka (2010) [11] was non-significant. A more detailed analysis revealed that the weak correlation originated from a very low correlation between arousal ratings for the positive nouns. This finding is not easily explained in view of the significant positive correlations with the arousal ratings for the positive words in ANEW [14] and Redondo et al. (2007) [12]. A possible explanation might be that our database had only 20 positive words in common with that of Eilola and Havelka (2010) [11], compared to 68 with ANEW [14] and Redondo et al. (2007) [12]. Another possibility could be the different conceptualizations of the arousal scale, but this explanation seems less probable because the arousal scale used by Eilola and Havelka (2010), a Likert-scale ranging from 0 to 9 without a ‘neutral’ midpoint [11], was more reminiscent of the SAM scale [30], which was used in both ANEW [14] and Redondo et al. (2007) [12], than our scale was. Also, a more detailed analysis of the arousal ratings for common single positive words revealed near-opposite ratings for some of these words. For example, the mean arousal rating of the word ‘adventure’ (*seikkailu*) was 5.09 on a scale from 1 to 7 in our study, rendering it a classification as high-arousing, whereas it elicited a mean arousal rating of 3.91 in the Eilola and Havelka (2010) study [11].

Note that all the correlations between arousal ratings in the different languages were weaker compared to the correlations between the valence ratings. This finding has also been observed in other studies comparing ratings between different languages [11]–[13], and has been taken to suggest culture-specific differences in emotional reactivity to words.

The expectation that we would find age- and gender-related differences in valence and arousal ratings for these nouns was confirmed, but support for an age-related positivity effect in the valence ratings was only partial. We found a trend indicating that older adults gave slightly more positive mean ratings than middle-aged adults. Although this trend can be taken as support for the positivity effect, it contradicts the finding in the study by Gilet et al. (2012), in which middle-aged adults gave more positive mean ratings compared to the other age groups [18]. The reason for this discrepancy may be due to the difference in the age group composition between our study and that of Gilet et al. (2012) [18]. As expected, and offering further support for a positivity effect, as well as in line with the findings of Grühn and Smith (2008) [15], older (and middle-aged) adults in our study gave more positive ratings for the positive nouns compared to young adults (and adolescents). Partially in line with the study by Keil and Freund (2009) [22], middle-aged and older adults rated the negative nouns as more negative than adolescents and young adults. Taken together, middle-aged and older adults gave more extreme valence ratings in both ends of the valence scale compared to adolescents and young adults in our study.

The age-related effects on arousal ratings partially supported our predictions. However, they were mostly in contrast to previous research, which might be due to the inclusion of a fourth age group (adolescents) in our study or to the different operationalizations of the arousal scale. Partially in line with previous research [18] and in partial support of our predictions, older adults gave higher mean arousal ratings than adolescents and middle-aged adults. However, adolescents gave lower mean arousal ratings compared to all the other age groups. In partial support of the findings by Keil and Freund (2009) [22] as well as of our expectations, but in contrast to the findings by Grühn and Smith (2008) [15], older adults rated the negative nouns as more arousing than adolescents and middle-aged adults. Note, however, that the adolescents rated the negative nouns as less arousing all the other age groups, as did the middle-aged adults compared to both young and older adults. Regarding arousal ratings for neutral nouns, older adults gave equally high mean ratings as young adults, but higher than adolescents and middle-aged adults, which was in partial agreement with previous work by Grunwald et al. (1999) [21]. Contrary to the findings by Grühn and Smith (2008) [15] there were no age-related differences in arousal ratings for the positive nouns.

The null effect of gender on valence and arousal ratings reported by Gilet et al. (2012) [18] and Redondo et al. (2007) [12] was not confirmed. As predicted, and in line with previous research suggesting a stronger reactivity to emotion-laden stimuli in women [13], [23], women gave more extreme mean valence ratings in both ends of the scale. Women also rated the neutral nouns as more positive in our study. Gender-related effects were modified by age for the valence ratings such that older women gave more positive mean ratings than older men. An interaction was also observed for the valence ratings of the neutral and the negative nouns, so that adolescent women gave more negative ratings for the negative nouns compared to adolescent men, and young as well as older adult women gave more positive ratings for the neutral nouns. Regarding the positive nouns, women gave more positive ratings than men in each age group.

In support for the contention of stronger and more intense reactivity to stimuli in women and in line with the findings of Soares et al. (2012) [13], women gave higher mean arousal ratings than men. In agreement with our expectations and in partial agreement with the study by Grunwald et al. (1999) [21], women rated the negative and the neutral, but not the positive nouns, as more arousing than men. This finding emerged in every age group. To our knowledge, the present study is the first to report that gender effects varied as a function of age for valence and arousal ratings of written words.

The effect sizes for the statistically significant age- and gender-related differences in valence and arousal ratings were rather small. This has been observed in previous studies as well (e.g., [15], [23]). It thus seems that the demographic characteristics of the raters were of lesser importance than the inherent affective properties of the nouns themselves. This in its turn suggests that the language- and culture-specificity of valence and arousal ratings of words might be a more important aspect than the demographic characteristics of the raters to consider when establishing norms for the affective properties of words. This finding is further supported by the fact that there was a discrepancy between the strength of the correlation for valence and arousal ratings between databases for different languages. The correlation for valence ratings was stronger than for arousal ratings, confirming the findings in previous work [11]–[13].

### Limitations of the Present Study

Using a web survey as a method to collect data has both benefits and drawbacks. A web survey enables the gathering of a large public sample of raters in a time- and cost-efficient way. We aimed at ensuring that the questionnaire met the requirements presented by Dillman, Tortora, and Bowker (1998), which have been developed in order to minimize errors of sampling, coverage, measurement, and non-response [46]. However, the problem of coverage, that is, non-response due to lack of computer skills or access to computers [47], was still encountered in this study, particularly regarding the older adults. The age range in this study was 16 to 77 years, but mean age was only 33 years. The problem surfaced despite the fact that we sent the e-mail invitations to organizations for elderly adults and to as heterogeneous demographic groups as possible in order to reach a maximally representative sample. This problem also pertains to the generalizability of our findings, as for example the age and gender distributions of our sample do not represent that of the general population in Finland at the time of data collection.

The computer program used to develop the questionnaires also had some limitations. It was impossible to prevent multiple responses from the same individual and to check for completeness, which resulted in a large amount of missing values. It was also impossible to calculate response rates or view rates, participation rates, and completion rates, which have been suggested as replacement for the calculation of response rates in internet surveys by Eysenbach (2004) [43]. However, our data was carefully screened for multiple responses and missing values, and seemingly unreliable cases were removed from the statistical analyses.

### Conclusion and Future Directions

In conclusion, the study showed that the strongest effects on the valence and arousal ratings were observed for the words’ inherent affective properties, suggesting that language- and culture-specificity carries more weight for the establishment of affective norms for words than the age or gender of the raters. Still, we found some age- and gender-related effects on the valence and arousal ratings, thus demonstrating the utility of providing age- and gender-specific normative data to enable the control and manipulation of between-subject variation in the cognitive processing of stimuli.

It would be interesting to extend this research to studying effects of demographic characteristics of raters on discrete emotion ratings of words. Providing discrete emotion norms for words further enlarges the field of applicability of databases containing norms for affective stimulus properties [48]-[49]. Furthermore, in light of previous research [50] it would be important to collect concreteness or imageability ratings for these words.

In conclusion, we hope that the present database, which includes valence and arousal ratings as well as objective psycholinguistic estimates for the stimulus words, provides a stimulus source for further studies on word processing in Finnish.

## Supporting Information

Database S1 **Affective ratings and psycholinguistic measures for 420 Finnish nouns.** The database includes valence and arousal ratings separately for the whole sample (All) and for the demographic groups divided by gender and age, as well as psycholinguistic measures obtained by the WordMill Lexical Search Program [42] from an unpublished extensive database of written Finnish, and additional information on each word (English translation, number of word list in which the word appeared). The contents of the database are described in more detail in the Description of the Supplementary Material section in the article.
(XLS)
